# Bifunctional DEGS2 has higher hydroxylase activity toward substrates with very-long-chain fatty acids in the production of phytosphingosine ceramides

**DOI:** 10.1016/j.jbc.2023.104603

**Published:** 2023-03-11

**Authors:** Ai Ota, Hiroya Morita, Tatsuro Naganuma, Masatoshi Miyamoto, Keisuke Jojima, Koki Nojiri, Junko Matsuda, Akio Kihara

**Affiliations:** 1 Faculty of Pharmaceutical Sciences, Hokkaido University, Sapporo, Japan; 2 Department of Pathophysiology and Metabolism, Kawasaki Medical School, Okayama, Japan

**Keywords:** bifunctional enzyme, ceramide, enzyme mechanism, epidermis, lipid, permeability, sphingolipid

## Abstract

Phytosphingosine (PHS) is a sphingolipid component present mainly in epithelial tissues, including the epidermis and those lining the digestive tract. DEGS2 is a bifunctional enzyme that produces ceramides (CERs) containing PHS (PHS-CERs) *via* hydroxylation and sphingosine-CERs *via* desaturation, using dihydrosphingosine-CERs as substrates. Until now, the role of DEGS2 in permeability barrier functioning, its contribution to PHS-CER production, and the mechanism that differentiates between these two activities have been unknown. Here, we analyzed the barrier functioning of the epidermis, esophagus, and anterior stomach of *Degs2* KO mice and found that there were no differences between *Degs2* KO and WT mice, indicating normal permeability barriers in the KO mice. In the epidermis, esophagus, and anterior stomach of *Degs2* KO mice, PHS-CER levels were greatly reduced relative to WT mice, but PHS-CERs were still present. We obtained similar results for *DEGS2* KO human keratinocytes. These results indicate that although DEGS2 plays a major role in PHS-CER production, another synthesis pathway exists as well. Next, we examined the fatty acid (FA) composition of PHS-CERs in various mouse tissues and found that PHS-CER species containing very-long-chain FAs (≥C21) were more abundant than those containing long-chain FAs (C11–C20). A cell-based assay system revealed that the desaturase and hydroxylase activities of DEGS2 toward substrates with different FA chain lengths differed and that its hydroxylase activity was higher toward substrates containing very-long-chain FAs. Collectively, our findings contribute to the elucidation of the molecular mechanism of PHS-CER production.

Sphingolipids are multifunctional lipids that are present in eukaryotes and some prokaryotes (1). In eukaryotes, together with glycerophospholipids and sterols, they constitute biological membranes. They are enriched in the plasma membrane and form lipid microdomains that serve as a platform for the proteins involved in signal transduction (2, 3). Ceramides (CERs), the hydrophobic backbone of complex sphingolipids, form a multilayered lipid structure (the lipid lamellae) in the epidermal stratum corneum and are important for the permeability barrier function of the skin (skin barrier) (1).

Complex sphingolipids are composed of a CER and a polar head group. The polar head group in mammals is a phosphocholine or sugar chain, and the complex sphingolipids that contain these are called sphingomyelins (SMs) and glycosphingolipids, respectively (1). The structure of CERs is a long-chain base (LCB) and a fatty acid (FA) linked *via* an amino bond. Mammals have five LCBs (sphingosine [SPH], dihydrosphingosine [DHS], phytosphingosine [PHS], 6-hydroxysphingosine, and 4,14-sphingadiene), which commonly have hydroxyl groups at positions 1 and 3 and an amino group at position 2 (1, 4). LCBs can also be represented by their number of hydroxyl groups (d, di; t, tri), carbon chain length, and the number of double bonds. For example, DHS, SPH, and PHS with a carbon chain length of 18 are represented by d18:0, d18:1, and t18:0, respectively. Hereafter, we use the d/t notation to indicate the chain length of LCBs to distinguish them from FAs, for which we used the C notation to indicate chain length (*e.g.*, stearic acid, C18:0). The hydroxyl and amino groups in sphingolipids act as donors and acceptors of hydrogen bonds and enhance lipid–lipid interactions, facilitating the formation of lipid microdomains or stable lipid lamellae in the stratum corneum.

FAs are classified into long-chain FAs (LCFAs), which have C11–C20, and very-long-chain FAs (VLCFAs), with ≥C21. The FA portions of most glycerophospholipids are saturated, monounsaturated, or polyunsaturated LCFAs with C16–C20 (5). In contrast, the constituent FAs of the sphingolipids in most tissues are C16:0–C24:0 saturated FAs, with the addition of C24:1 monounsaturated FA (6, 7, 8). The percentage of VLCFA-containing sphingolipids varies from 20 to 80%, depending on the tissue (6, 7, 8).

The *de novo* biosynthesis pathway of sphingolipids begins with the condensation of serine and acyl-CoA to produce 3-ketodihydrosphingosine, followed by the production of DHS *via* the reduction of 3-ketodihydrosphingosine (1) (Fig. 1). DHS is then converted to CER containing DHS (DHS-CER) by CER synthase, using DHS and acyl-CoA as substrates. In many tissues, most DHS-CER is subsequently converted into SPH-containing CER (SPH-CER) by introducing a double bond between C4 and C5 of the DHS portion. This reaction is primarily catalyzed by the DHS-CER Δ4-desaturase DEGS1 (9). In contrast, in epithelial tissues such as the epidermis, those lining the digestive tract, and kidney, PHS-containing CER (PHS-CER) is produced *via* the 4-hydroxylation of DHS-CER by the DHS-CER 4-hydroxylase DEGS2 (10, 11). DEGS2 is a bifunctional enzyme with Δ4-desaturase activity in addition to its 4-hydroxylase activity (9). Therefore, some of the SPH-CERs in mammals may be produced by DEGS2. In *Degs1* KO mice, SPH-CER levels are greatly reduced relative to WT mice, but SPH-CERs are not entirely absent (12, 13). It is likely that these SPH-CERs are produced by DEGS2.

**Figure 1:**
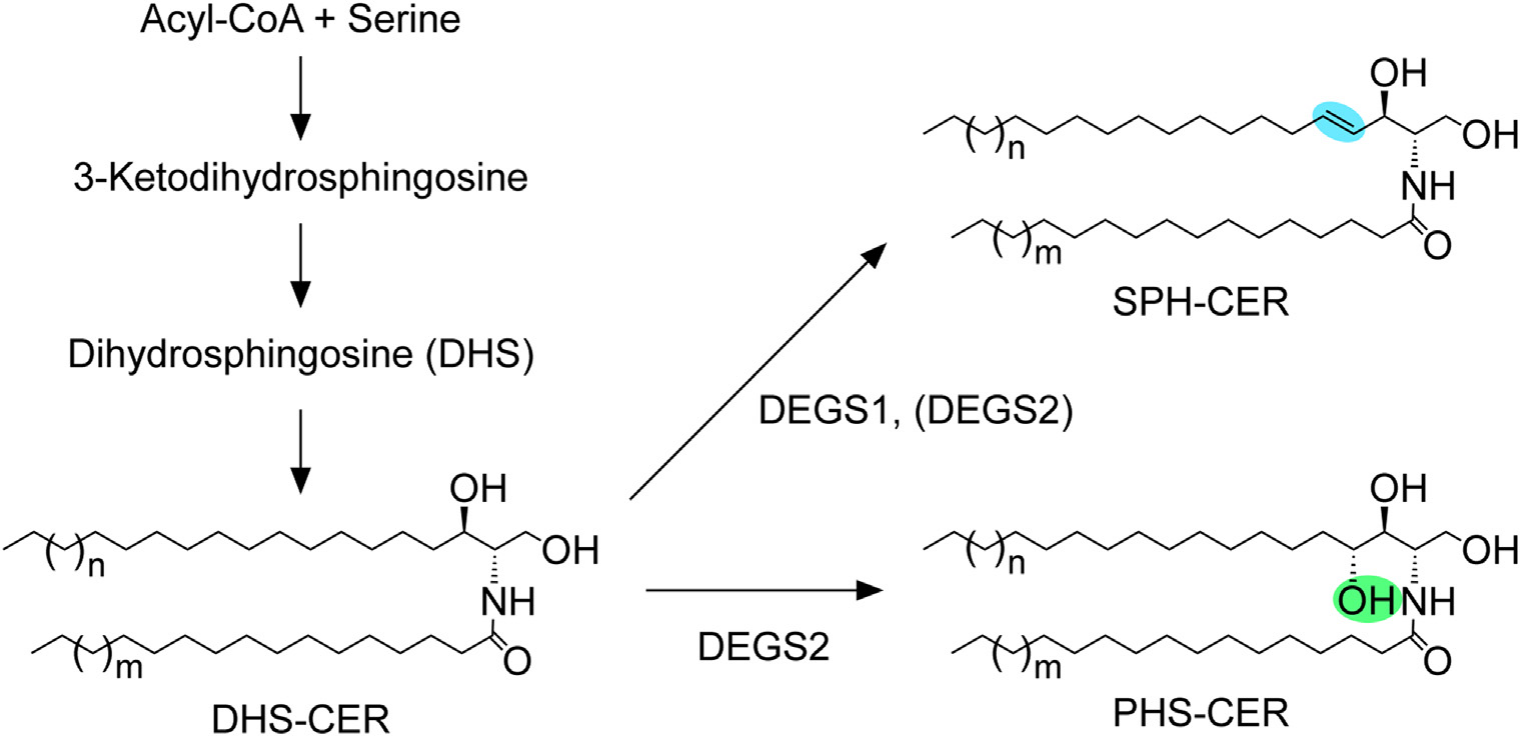
**Synthesis pathways of SPH-CER and PHS-CER.** Biosynthesis of sphingolipids/CERs begins with the condensation of acyl-CoA and serine, followed by the production of 3-ketodihydrosphingosine, DHS, and DHS-CER. DHS-CER is converted to SPH-CER mainly by DEGS1 and partly by DEGS2. Alternatively, DHS-CER is converted to PHS-CER by DEGS2. CER, ceramide; DHS-CER, DHS containing CER; PHS-CER, PHS-containing CER; SPH-CER, SPH-containing CER.

The major components of epidermal lipid lamellae are CERs, cholesterol, and free FAs. The epidermis contains a large number and variety of CERs, of which PHS-CERs are the most abundant in humans (14, 15). PHS-CERs have one more hydroxyl group than DHS-CERs and SPH-CERs. Thus, PHS-CERs can form more hydrogen bonds between lipids, enhancing the permeability barrier function of epithelial tissues, including epidermis. However, the role of PHS-CERs in the permeability barrier function has not been experimentally proven using *Degs2* KO mice. In addition, it is unclear whether DEGS2 differentially exhibits hydroxylase or desaturase activity depending on the carbon chain length of its substrates, DHS-CERs. Therefore, in this study, we investigated the role in permeability barrier functioning of PHS-CERs using *Degs2* KO mice and the molecular mechanism that differentiates between the hydroxylase and desaturase activities of DEGS2 using a cell-based assay system.

## Results

### Skin barrier function is normal in *Degs2* KO mice

**Figure 2:**
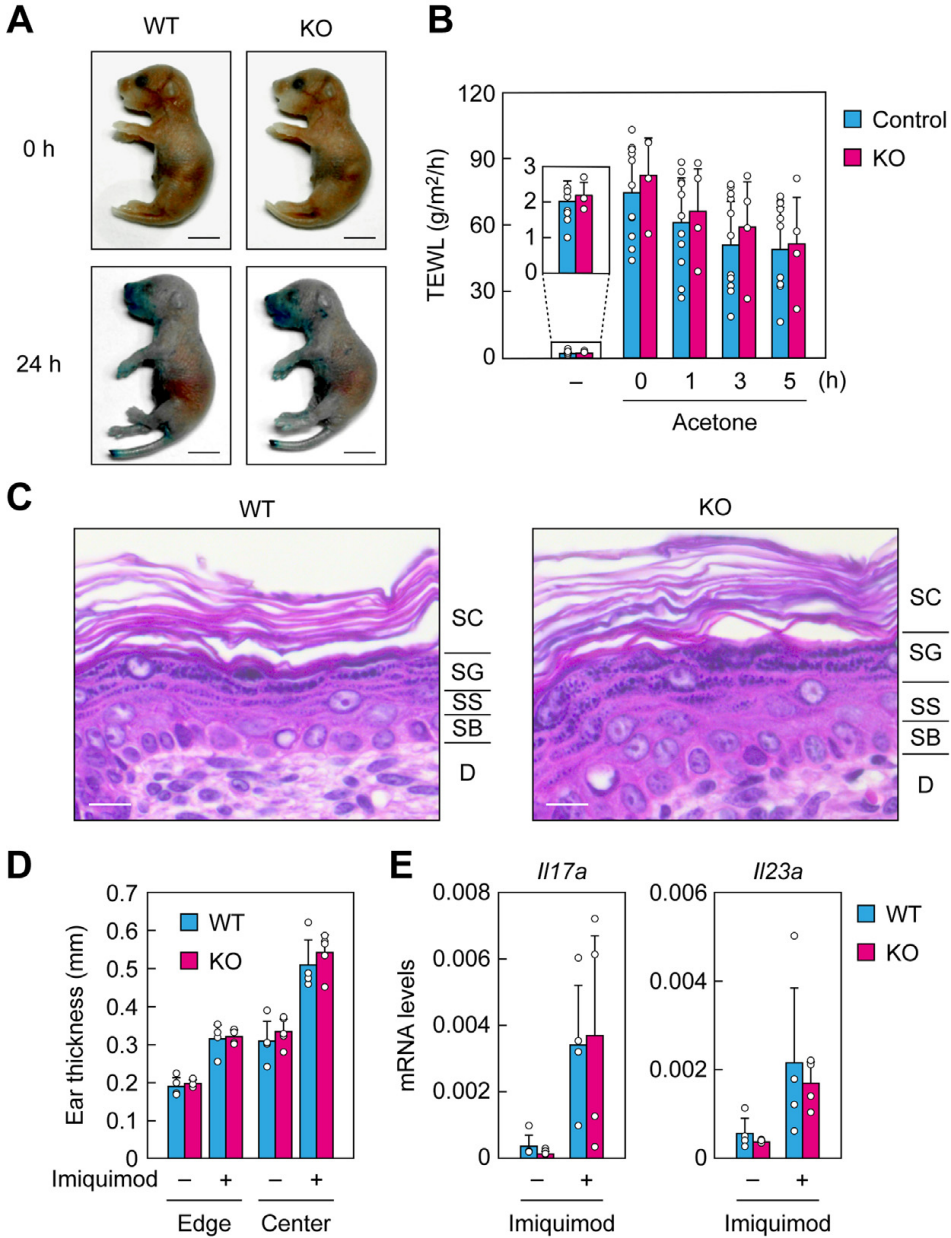
**Normal skin barrier of *Degs2* KO mice.***A*, WT and *Degs2* KO mice were stained with toluidine blue for 0 or 24 h and photographed. The scale bars represent 5 mm. *B*, control (n = 11; WT [n = 2] and *Degs2* heterozygous KO [n = 9]) or *Degs2* KO (n = 4) mice at postnatal days 0 to 2 were subjected to TEWL measurement before or 0, 1, 3, or 5 h after acetone treatment. Values presented are means + SD. *C*, paraffin sections of postnatal day 0 WT and *Degs2* KO mouse skin were prepared and stained with hematoxylin and eosin, and bright field images were obtained. The scale bars represent 10 μm. *D* and *E*, imiquimod-containing cream or control hydrophilic cream were applied to the right and left ears of WT (n = 4) and *Degs2* KO (n = 4) mice every day for 4 days. *D*, the thickness of the edge and center of the ears was measured with a caliper. Values presented are means + SD. *E*, total RNA was prepared from the ears and subjected to quantitative real-time RT-PCR using *Il17a*-, *Il23a*-, and *Gapdh*-specific primers. Values presented are mean + SD mRNA levels relative to *Gapdh*. D, dermis; SB, stratum basale; SC, stratum corneum; SG, stratum granulosum; SS, stratum spinosum; TEWL, transepidermal water loss.

To clarify the role of PHS-CERs in skin barrier formation, we analyzed *Degs2* KO mice. First, to investigate the skin barrier function under normal conditions, we performed toluidine blue staining of WT and *Degs2* KO mice and measured their transepidermal water loss (TEWL). Mice with reduced skin permeability barrier function are stained darker by toluidine blue and have higher TEWL values (16, 17, 18). We found no significant differences between the WT and *Degs2* KO mice in either analysis (Fig. 2, *A* and *B*). Next, we examined their skin barrier function (by measuring TEWL) and the time course of its recovery under conditions in which the skin barrier was impaired by acetone treatment. When the skin of control mice was treated with acetone, TEWL increased approximately 38-fold and then recovered slowly (Fig. 2*B*). The TEWL value and the time course of its recovery after acetone treatment in KO mice were comparable to those of control mice.

CERs are one of the major components of stratum corneum lipid lamellae, which are important structures for skin barrier function (1). Patients with ichthyosis, a skin disorder involving reduced skin barrier function, exhibit hyperkeratosis (increased number of stratum corneum layers) and impaired lipid lamella formation (19). Similar morphological changes are observed in ichthyosis model mice (*e.g.*, the mice are unable to produce acylceramides [acyl-CERs], specialized CERs for skin barrier) (16, 17, 18, 20). We performed hematoxylin/eosin staining to examine the number of stratum corneum layers and lipid lamella formation. In the stratum corneum of WT mice, we observed approximately ten layers and gaps between the corneocytes (corresponding to lipid lamellae), as previously reported (16, 17, 18) (Fig. 2*C*). The morphology of the stratum corneum of *Degs2* KO mice did not differ from that of WT mice. We also observed no differences in the morphology of the other layers of the epidermis (stratum granulosum, stratum spinosum, or stratum basale).

Levels of PHS-CERs are reduced in patients with psoriasis (21, 22). We next examined the effects of *Degs2* disruption on psoriasis pathology using a psoriasis model induced by imiquimod. When imiquimod was applied to the ears of WT mice, ear thickness and the expression of interleukin-17 (*Il17a*) and interleukin-23 (*Il23a*) were increased (Fig. 2, *D* and *E*). Similar results were obtained for *Degs2* KO mice. These results indicate that the skin barrier formation of *Degs2* KO mice is normal.

### PHS-CERs are reduced but remain in the epidermis of *Degs2* KO mice

**Figure 3:**
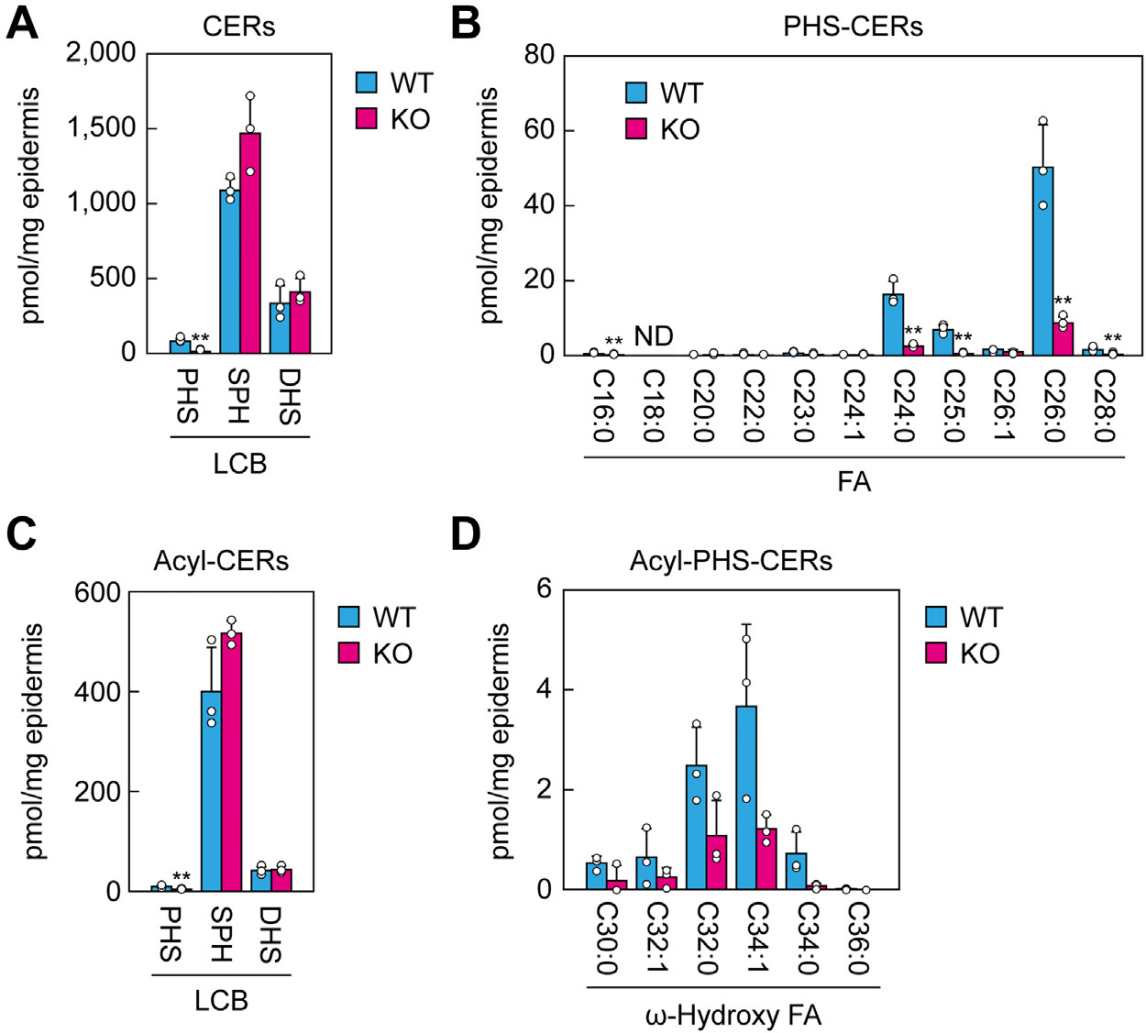
**Greatly reduced but residual presence of PHS-containing CERs in the epidermis of *Degs2* KO mice.** Lipids were extracted from epidermis of postnatal day 0 WT (n = 3) and *Degs2* KO (n = 3) mice, and CERs (*A* and *B*) and acyl-CERs (*C* and *D*) were measured *via* LC-MS/MS. Values presented are means + SD (∗∗*p* < 0.01; Student’s *t* test) of the total quantities of PHS-CERs, SPH-CERs, and DHS-CERs (*A*); the quantity of each PHS-CER species containing the indicated FA moiety (*B*); the total quantities of acyl-PHS-CERs, acyl-SPH-CERs, and acyl-DHS-CERs (*C*); and the quantity of each acyl-PHS-CER species containing the indicated ω-hydroxy FA (*D*). CER, ceramide; DHS-CER, DHS containing CER; FA, fatty acid; PHS-CER, PHS-containing CER; SPH-CER, SPH-containing CER.

To investigate why *Degs2* KO mice did not show skin barrier abnormalities, we examined epidermal CER composition using LC coupled with MS/MS. We found that the levels of PHS-CERs in *Degs2* KO epidermis were reduced to 16.7% of those in WT mice but not completely lost (Fig. 3*A*). In WT mice, the PHS-CERs included several species differing in FA moiety, and VLCFA-containing species, especially C26:0 and C24:0 species, were abundant (Fig. 3*B*). In *Degs2* KO mice, all PHS-CER species were reduced, regardless of their FA moiety. We next examined the levels of acyl-CERs (acyl-PHS-CERs, acyl-SPH-CERs, and acyl-DHS-CERs), which are composed of an LCB, an ω-hydroxy FA, and linoleic acid. The levels of acyl-PHS-CERs in the *Degs2* KO epidermis were reduced to 33% of those in WT mice (Fig. 3*C*). The chain lengths of the ω-hydroxy FA moiety of acyl-PHS-CERs were C30–C36, and the quantities of acyl-PHS-CER species with all FA chain lengths were reduced to a similar extent (Fig. 3*D*). In contrast, the quantities of SPH-CERs, DHS-CERs, and their acyl forms were not reduced in the epidermis of *Degs2* KO mice relative to WT mice (Fig. 3, *A* and *C*).

### PHS-CERs are reduced but remain in *DEGS2* KO human keratinocytes

**Figure 4:**
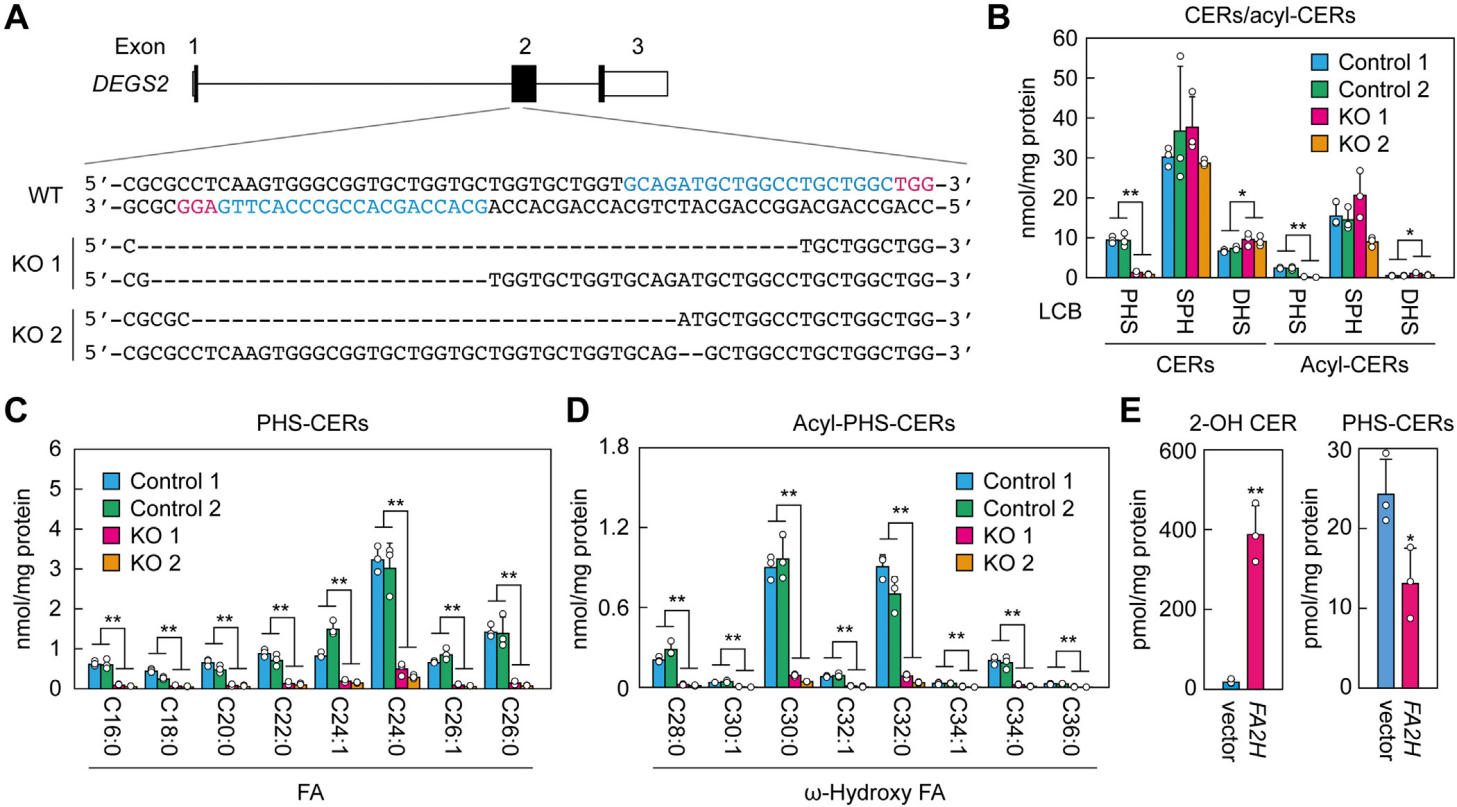
**Greatly reduced but residual presence of PHS-containing CERs in *DEGS2* KO keratinocytes.***A*, *DEGS2* KO keratinocytes were generated using the CRISPR/Cas9 system. The exon structure (*black*, coding sequence; *white*, untranslated regions) of human *DEGS2* and the nucleotide sequences of WT and *DEGS2* KO keratinocytes (KO clones 1 and 2) around the guide RNA target sequences (*light blue*) and the protospacer-adjacent motif sequences (*magenta*) in exon 2 are shown. *B*–*D*, lipids were extracted from controls (controls 1 and 2) and *DEGS2* KO keratinocytes (KO 1 and 2) differentiated for 14 days, and CERs and acyl-CERs were analyzed *via* LC-MS/MS. Values presented are means + SD (n = 3; ∗∗*p* < 0.01; ∗*p* < 0.05; Scheffé’s test) of the total quantities of PHS-CERs, SPH-CERs, DHS-CERs, acyl-PHS-CERs, acyl-SPH-CERs, and acyl-DHS-CERs (*B*); the quantity of each PHS-CER species containing the indicated FA moiety (*C*); and the quantity of each acyl-PHS-CER species containing the indicated ω-hydroxy FA (*D*). *E*, HEK 293T cells were transfected with pCE-puro 3× FLAG-1 (vector) or pCE-puro 3× FLAG-FA2H plasmid. After 24 h of transfection, lipids were extracted, and 2-hydroxy palmitic acid-containing SPH-CER (2-OH CER) and PHS-CERs were quantified *via* LC-MS/MS. Values presented are means + SD (n = 3; ∗∗*p* < 0.01; ∗*p* < 0.05; Student’s *t* test). CER, ceramide; DHS-CER, DHS containing CER; PHS-CER, PHS-containing CER; SPH-CER, SPH-containing CER.

CER composition differs between human and mouse stratum corneum, with much lower quantities of PHS-CERs and acyl-PHS-CERs in mice than in humans (14). Therefore, we next generated *DEGS2* KO keratinocytes *via* the CRISPR/Cas9 system using human immortalized keratinocytes to investigate the role of *DEGS2* in the production of PHS-CERs and acyl-PHS-CERs in humans. To avoid off-target effects, we used a nickase-type Cas9 for the construction. We obtained two independent *DEGS2* KO clones with deletions in exon 2: the first clone (KO 1) had deletions of 49 bp and 25 bp, and the second clone (KO 2) had deletions of 36 bp and 2 bp (Fig. 4*A*). These two KO clones and a further two control clones were differentiated, and we measured their CERs *via* LC-MS/MS. Both of the KO clones had greatly reduced levels of PHS-CERs and acyl-PHS-CERs relative to the control clones (PHS-CERs, 9.0–14.6% of controls; acyl-PHS-CERs, 4.1–10.0% of controls; Fig. 4*B*). The differentiated keratinocytes contained PHS-CERs with C16–C26 FAs and acyl-PHS-CERs with C28–C36 ω-hydroxy FAs (Fig. 4, *C* and *D*). All species of PHS-CERs and acyl-PHS-CERs were reduced to similar extents in the KO keratinocytes relative to the controls. On the other hand, there were no differences in the levels of SPH-CERs or acyl-SPH-CERs in the KO keratinocytes relative to the controls, while those of DHS-CERs and acyl-DHS-CERs were slightly higher (Fig. 4*B*).

With respect to the reasons for the fact that some PHS-CERs remained in *DEGS2* KO keratinocytes, we could not rule out the possibility that PHS-CERs present in the culture medium had been incorporated into the KO keratinocytes. To test this possibility, we assessed the quantity of PHS-CERs in the medium *via* LC-MS/MS and found that 1 ml of medium contained 0.3 fmol of PHS-CERs. This was <1 × 10^−5^ of the quantity present in the KO keratinocytes, so it could not have been the source of the PHS-CERs that remained in the KO keratinocytes. We also measured the quantity of PHS—the precursor of PHS-CERs—in the medium, but it was below the detection limit.

Another possible mechanism of PHS-CER production was that 2-hydroxy acyl-CoA, rather than acyl-CoA, may be used as a substrate by serine palmitoyltransferase (SPT) in the first reaction of the sphingolipid biosynthesis pathway, which would lead to PHS production in the subsequent reduction reaction. To test this possibility, we overproduced the FA 2-hydroxylase FA2H, which produces 2-hydroxy FAs (23), in HEK 293T cells. Overexpression of FA2H increased the quantity of SPH-CERs containing 2-hydroxy FA, confirming that 2-hydroxy FAs were produced as expected (Fig. 4*E*). However, PHS-CER levels were not increased by FA2H overproduction but were actually reduced relative to the vector control. These results indicate that 2-hydroxy acyl-CoA is not used as a precursor of PHS in the *de novo* sphingolipid synthesis pathway. The PHS-CERs that were present in the *Degs2* KO mice were therefore probably produced by an unknown enzyme.

### Many PHS-CERs contain VLCFAs

**Figure 5:**
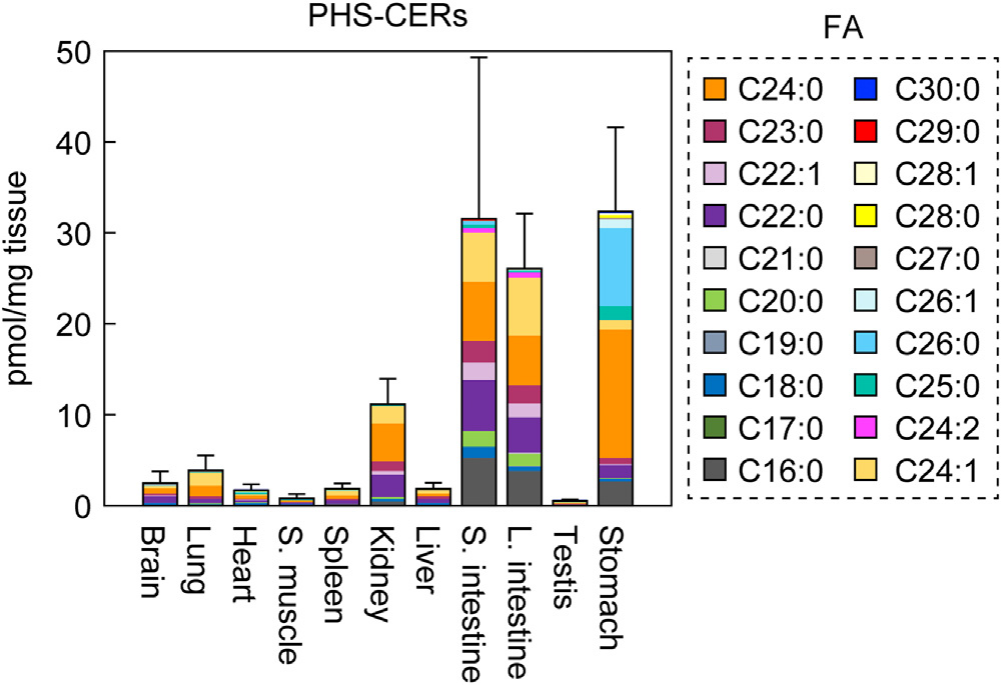
**Tissue distribution of PHS-CERs.** Lipids were extracted from 11 tissues (brain, lung, heart, skeletal muscle, spleen, kidney, liver, small intestine, large intestine, testis, and stomach) of 4-month-old female mice (n = 3), and the quantities of PHS-CERs were examined *via* LC-MS/MS. Values presented are means + SD of the total quantities of PHS-CERs, with FA species color coded. CER, ceramide; FA, fatty acid; PHS-CER, PHS-containing CER.

Although it is known that PHS-CERs are abundant in epithelial tissues such as the epidermis, those lining the digestive tract, and the kidney (14, 24, 25), the detailed PHS-CER species composition in a wide range of tissues remains largely unclear. In this study, we therefore analyzed the quantities and composition of PHS-CERs in 11 mouse tissues (brain, lung, heart, skeletal muscle, spleen, kidney, liver, small intestine, large intestine, testis, and stomach) *via* LC-MS/MS. We excluded the epidermis because we had already analyzed it (Fig. 3). We found that PHS-CERs were abundant in the small intestine, stomach, and large intestine and somewhat less so in the kidney (Fig. 5). Only small quantities were present in the brain, lung, heart, skeletal muscle, spleen, liver, and testis. The most abundant PHS-CER species in the small intestine and kidney was C24:0, followed by C22:0 and C24:1. The order of abundance of PHS-CER species in the large intestine and stomach was C24:1 > C24:0 > C22:0 and C24:0 > C26:0 > C16:0, respectively. The PHS-CERs containing VLCFAs were thus more abundant than those containing LCFAs, and they constituted the following percentages of total PHS-CERs in these tissues: small intestine, 74%; stomach, 91%; large intestine, 78%; and kidney, 92%. These values are higher than our previously reported percentages of SPH-CERs containing VLCFAs (*e.g.*, 40% in the small intestine and 58% in the kidney) (8).

### DEGS2 prefers to use VLC substrates for hydroxylation reaction than desaturation reaction

**Figure 6:**
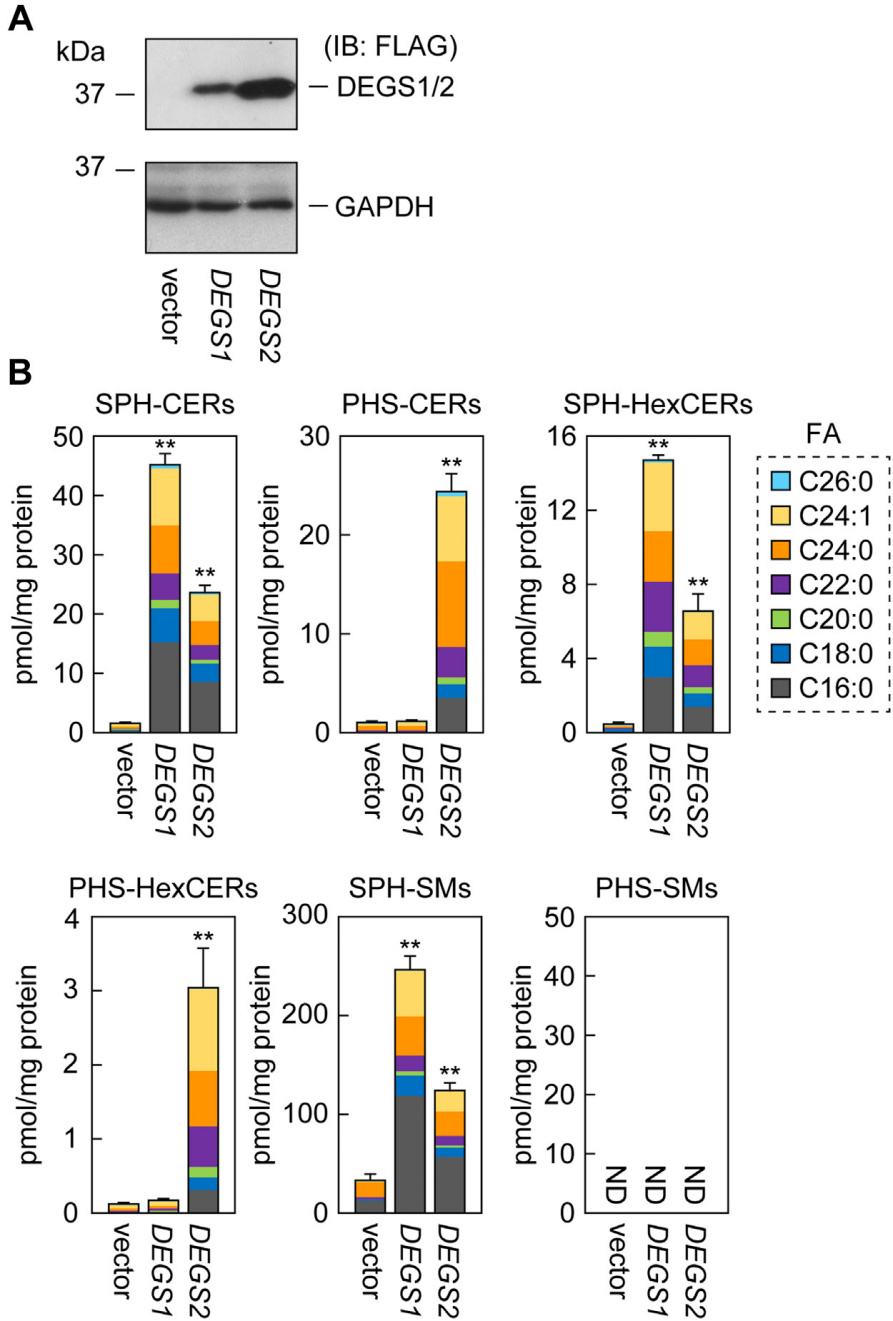
**Preference for VLC substrates in the hydroxylation reaction by DEGS2.***A* and *B*, *DEGS1* KO HAP1 cells were transfected with pEFh-3× FLAG-1 (vector), pEFh-3× FLAG-DEGS1, or pEFh-3× FLAG-DEGS2 plasmid. After 24 h, cells were labeled with 2 μM *d*_7_-DHS for 4 h. *A*, proteins were prepared and subjected to immunoblotting (IB) using anti-FLAG or anti-GAPDH (loading control) antibodies. *B*, lipids were extracted, and *d*_7_-labeled SPH-CERs, PHS-CERs, SPH-HexCERs, PHS-HexCERs, SPH-SMs, and PHS-SMs were quantified *via* LC-MS/MS. Values presented are means + SD of the respective lipids, with FA species color-coded (n = 3; ∗∗*p* < 0.01; Dunnett’s test *versus* vector control). CER, ceramide; DHS-CER, DHS containing CER; FA, fatty acid; HexCER, monohexosylceramide; ND, not detected; PHS-CER, PHS-containing CER; SM, sphingomyelin; SPH-CER, SPH-containing CER.

To clarify the reason for the abundance of the VLC species of PHS-CERs, we examined the substrate specificity of DEGS2, a bifunctional enzyme exhibiting both hydroxylase and desaturase activities. In general, mammalian cells have high DHS-CER desaturase activity exerted by endogenous DEGS1. Therefore, even if DEGS2 is overexpressed in mammalian cells, its desaturase activity is expected to be masked and cannot be accurately measured. To circumvent this problem, we generated *DEGS1* KO cells *via* the CRISPR/Cas9 system using myelogenous leukemia-derived human HAP1 cells, which are near-haploid and therefore facilitate the easy generation of KO cells. We overexpressed 3× FLAG-tagged DEGS1 or DEGS2 in the *DEGS1* KO cells. Immunoblot analysis showed that 3× FLAG-DEGS2 was more highly expressed than 3× FLAG-DEGS1 (Fig. 6*A*). We labeled these cells with seven deuterium (*d*_7_)-containing DHS (*d*_7_-DHS) and measured the resulting *d*_7_-SPH-CERs and *d*_7_-PHS-CERs *via* LC-MS/MS. The quantity of *d*_7_-SPH-CERs produced by DEGS1 was 1.9 times that produced by DEGS2 (Fig. 6*B*). Considering the lower expression levels of DEGS1 than DEGS2 (Fig. 6*A*), this means the desaturase activity of DEGS1 was much higher than that of DEGS2. The most abundant *d*_7_-SPH-CER species produced by DEGS1 was C16:0, followed by C24:1, and C24:0, and the percentage of *d*_7_-SPH-CERs containing VLCFAs was 54% (Fig. 6*B*). The FA composition of *d*_7_-SPH-CERs produced by DEGS2 was similar to that of DEGS1, with 48% of *d*_7_-SPH-CERs containing VLCFAs. However, *d*_7_-PHS-CERs were produced only in cells expressing DEGS2. The quantity of *d*_7_-PHS-CERs produced by DEGS2 was comparable to that of *d*_7_-SPH-CERs produced by DEGS2. Of the *d*_7_-PHS-CERs species produced, C24:0 was the most abundant, followed by C24:1. The percentage of *d*_7_-PHS-CERs containing VLCFAs produced by DEGS2 was 80%, much higher than that of *d*_7_-SPH-CERs (48%).

A possible reason for the difference in FA composition (percentage of VLCFA-containing species) between *d*_7_-SPH-CERs and *d*_7_-PHS-CERs produced by DEGS2 is differences in the metabolic flow of SPH-CERs and PHS-CERs to complex sphingolipids (glycosphingolipids and SMs) depending on the FA chain lengths. To test this possibility, we measured the quantities of *d*_7_-monohexosylceramides (the simplest glycosphingolipids; HexCERs) and *d*_7_-SMs. The levels of *d*_7_-SPH-HexCERs and *d*_7_-SPH-SMs produced in DEGS1-overexpressing cells were 2.3- and 2.0-fold higher, respectively, than those produced in DEGS2-overexpressing cells, which correlated well with the differences in the levels of *d*_7_-SPH-CERs produced (2.1-fold; Fig. 6*B*). In DEGS2-overexpressing cells, *d*_7_-PHS-HexCERs were present, but we did not detect *d*_7_-PHS-SMs, which was consistent with our previous finding that metabolism of PHS-CERs to glycosphingolipids is predominant over that to SMs (26, 27). The percentages of *d*_7_-SPH-HexCERs and *d*_7_-SPH-SMs containing VLCFAs in DEGS2-overexpressing cells were 41% and 55%, respectively, whereas that of *d*_7_-PHS-HexCERs containing VLCFAs was 80%. These results indicate that not only PHS-CERs but also PHS-containing complex sphingolipids have more VLC species than SPH-containing CERs/complex sphingolipids in DEGS2-overexpressing cells. Based on these findings, we conclude that DEGS2 uses VLCFA-containing substrates (DHS-CERs) preferentially for hydroxylation reactions rather than desaturation reactions.

### PHS-CER with t20:0 is abundant in the esophagus and anterior stomach

**Figure 7:**
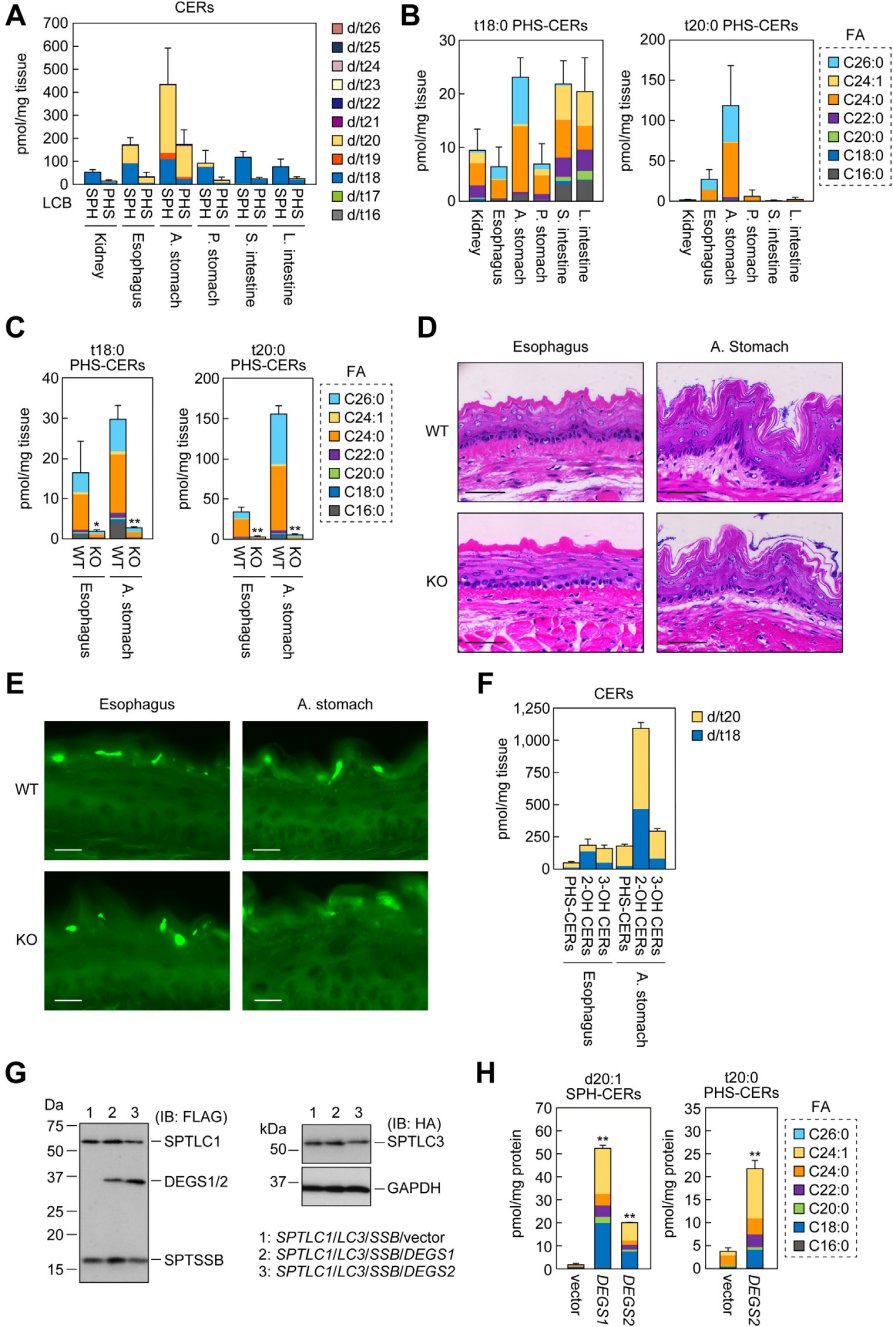
**Tissue distribution of t20:0 PHS-CERs and their production by DEGS2.***A* and *B*, lipids were extracted from six tissues (kidney, esophagus, anterior stomach, posterior stomach, small intestine, and large intestine) of 2-month-old WT mice (n = 3), and the quantities of SPH-CERs and PHS-CERs containing LCBs with chain lengths from d/t16 to d/t26 were quantified *via* LC-MS/MS. Values presented are means + SD of the total quantities of SPH-CERs and PHS-CERs (*A*; LCB chain lengths are color coded) and the quantities of t18:0- or t20:0-containing PHS-CERs (*B*; FA chain lengths are color-coded). *C*, lipids were extracted from the esophagus and anterior stomach of six-month-old WT (n = 3) and *Degs2* KO (n = 3) mice, and the quantities of t18:0 and t20:0 PHS-CERs were examined *via* LC-MS/MS. Values presented are means + SD of the total quantities of PHS-CERs, with FA species color coded. *D* and *E*, esophagus and anterior stomach of 6-month-old WT and *Degs2* KO mice were prepared and subjected to hematoxylin/eosin staining (*D*) and lucifer yellow assay (*E*). Scale bars, 50 μm (*D*) and 10 μm (*E*). *F*, lipids were extracted from the esophagus and anterior stomach of 6-month-old WT (n = 3) and *Degs2* KO (n = 3) mice, and PHS-CERs, SPH-CERs containing 2-hydroxy FA (2-OH CERs), and SPH-CERs containing 3-hydroxy FA (3-OH CERs) were quantified *via* LC-MS/MS. Values presented are means + SD of the total quantities of PHS-CERs, 2-OH CERs, and 3-OH CERs. *G* and *H*, *DEGS1* KO HAP1 cells were transfected with pCE-puro 3× FLAG-SPTLC1, pCE-puro 3× FLAG-SPTSSB, and pCE-puro HA-SPTLC3 plasmids together with pEFh-3× FLAG-1 (vector), pEFh-3× FLAG-DEGS1, or pEFh-3× FLAG-DEGS2 plasmid, and cultured for 24 h. *G*, proteins were prepared and subjected to immunoblotting (IB) with anti-FLAG, anti-HA, or anti-GAPDH (loading control) antibodies. *H*, lipids were extracted, and d20:1 SPH-CERs and t20:0 PHS-CERs were analyzed *via* LC-MS/MS. Values presented are means + SD of the total quantities of the respective lipids, with FA chain length color coded (n = 3; ∗∗*p* < 0.01; Dunnett’s test *versus* vector control for d20:1 SPH-CERs and Student’s *t* test for t20:0 PHS-CERs). CER, ceramide; DHS-CER, DHS containing CER; FA, fatty acid; PHS-CER, PHS-containing CER; SPH-CER, SPH-containing CER.

In the above analyses, we measured only CERs/complex sphingolipids with d/t18, which is the most common LCB chain length in mammals. However, the presence of sphingolipids with LCBs other than d/t18 has also been reported: for example, gangliosides (glycosphingolipids containing sialic acid) with d20:1 in brain, stomach, and intestinal mucosa, SMs with d16 to 20 LCBs in plasma, and CERs with d/t16 to 26 LCBs in stratum corneum (15, 28, 29, 30). Since the PHS chain-length diversity had thus far not been investigated, we next examined PHS chain lengths in the mouse tissues in which PHS-CERs were abundant (stomachs [anterior and posterior], small intestine, large intestine, and kidney; Fig. 5). In the posterior stomach, small intestine, large intestine, and kidney, most species were d/t18 for both SPH-CERs and PHS-CERs (Fig. 7*A*). In contrast, SPH-CERs and PHS-CERs with d/t20 were more abundant in the anterior stomach, accounting for 67% and 78% of the total SPH-CERs and PHS-CERs, respectively. The mouse stomach is divided into functionally distinct anterior and posterior stomachs, and the anterior stomach resembles the esophagus. Therefore, we also examined the LCB composition of CERs in the esophagus and found abundant d20:1 SPH-CERs (44% of total SPH-CERs) and t20:0 PHS-CERs (68% of total PHS-CERs). The most abundant FA moiety of t20:0 PHS-CERs was C24:0 in the anterior stomach, followed by C26:0 (Fig. 7*B*). In the t20:0 PHS-CERs of the esophagus, C24:0 and C26:0 species were present in comparable quantities. These FA compositions were similar to those of t18:0 PHS-CERs. These results indicate that the percentages of VLCFAs were high not only in t18:0 PHS-CERs but also in t20:0 PHS-CERs.

To investigate whether PHS-CERs remain in the esophagus and anterior stomach as they do in the epidermis in *Degs2* KO mice, we quantified PHS-CERs *via* LC-MS/MS. The quantities of t18:0 and t20:0 PHS-CERs in *Degs2* KO mice were much lower than those in WT mice (3.4–10.8%), but again were not entirely absent (Fig. 7*C*). Next, we examined the effects of the reduced PHS-CER levels in *Degs2* KO mice on the morphology and permeability barrier functioning of these tissues *via* hematoxylin/eosin staining and lucifer yellow assay, respectively. We found no differences in morphology or degree of lucifer yellow penetration between WT and *Degs2* KO mice (Fig. 7, *D* and *E*). One possible reason for the normal permeability barrier functioning in these tissues in *Degs2* KO mice is that there are also other hydroxy CER classes in addition to PHS-CERs that can form a permeability barrier. Indeed, LC-MS/MS analysis revealed that CERs with a 2-hydroxy FA or a 3-hydroxy FA were more abundant in the esophagus and anterior stomach of mice than PHS-CERs (Fig. 7*F*).

In the above substrate-specificity experiment (Fig. 6), we examined the bifunctional activities of DEGS2 toward d18:0 substrates; here we performed these analyses on d20:0 substrates. For this purpose, we overexpressed SPTLC1, SPTLC3, and SPTSSB, which encode SPT subunits, in *DEGS1* KO cells, together with DEGS1 or DEGS2. The chain lengths of the LCBs are defined by SPT, which catalyzes the first reaction in sphingolipid synthesis (15, 31). SPT is a hetero-oligomer composed of two large subunits (SPTLC1, -2, or -3) and one small subunit (SPTSSA or SPTSSB), and there are four SPT complexes: SPTLC1/2/SSA, SPTLC1/2/SSB, SPTLC1/3/SSA, and SPTLC1/3/SSB (31). Of these, SPTLC1/3/SSB can produce 3-ketodihydrosphingosines with a wide range of chain lengths (C16–C24) (15). We confirmed the expression of 3× FLAG-DEGS1, 3× FLAG-DEGS2, 3× FLAG-SPTLC1, HA-SPTLC3, and 3× FLAG-SPTSSB *via* immunoblotting (Fig. 7*G*). The most abundant d20:1 SPH-CER species produced by DEGS1 and DEGS2 were C24:1, followed by C18:0 (Fig. 7*H*). In contrast, C24:1 was the most abundant t20:0 PHS-CER species produced by DEGS2, followed by C24:0. The percentages of d/t20 CERs containing VLCFAs produced by DEGS2 were higher for PHS-CERs (78%) than for SPH-CERs (63%). Thus, DEGS2 exhibits a higher level of hydroxylase activity toward VLCFA-containing substrates than toward LCFA-containing substrates, regardless of whether the LCB chain length is d18 or d20.

## Discussion

CERs are a major component of lipid lamellae in the stratum corneum and are important for skin barrier function (1). PHS-CERs are the most abundant CERs in the human stratum corneum (14, 15). Levels of PHS-CERs are reduced in patients with atopic dermatitis and psoriasis (22, 32, 33), suggesting that PHS-CERs play a role in skin barrier formation and that these pathologies are caused by a deficiency in these CERs. In this study, we investigated the role of PHS-containing CERs (PHS-CERs and acyl-PHS-CERs) in skin barrier function using *Degs2* KO mice. These mice did not differ from WT mice in any of our analyses, whether under normal, artificially disrupted barrier, or psoriasis pathology conditions (Fig. 2). These results indicate that the skin barrier function of *Degs2* KO mice is normal. However, these results do not necessarily indicate that PHS-containing CERs do not contribute to skin barrier formation, especially in humans. While PHS-containing CERs are abundant in the human stratum corneum, they are present only in small quantities in the mouse stratum corneum: in humans, PHS-containing CERs constitute 35% of total CERs, whereas they constitute only 1% of those in mice (14). Instead, mice have abundant CERs with hydroxyl groups at other sites (3- or ω-position of the FA moiety) (14). In the present study, we also showed that the quantities of PHS-CERs were greatly reduced (but not entirely absent) in the esophagus and anterior stomach of *Degs2* KO mice relative to WT mice and that the permeability barrier functioning of these tissues of *Degs2* KO mice were normal (Fig. 7, *C*–*E*). The above discussion concerning the permeability barrier function of skin—that is, the small quantities of PHS-CERs and the compensation by other hydroxy CERs—is also applicable to these tissues.

Although the quantities of PHS-containing CERs were greatly reduced in *Degs2* KO mice and human *DEGS2* KO keratinocytes, PHS-CERs were not entirely absent in either case (Figs. 3, 4 and 7). The reason for this remains unclear, but there are several possibilities. First, the remaining PHS-containing CERs may have been derived from the diet (in the case of the mice) or the culture medium (in the case of the keratinocytes). However, this is highly unlikely. Although the quantities of PHS and PHS-CERs in the mouse diet are unknown, dietary PHS/PHS-CERs must be absorbed in the small intestine and then transported to other tissues, including the epidermis. In addition, PHS and PHS-CERs were barely detectable in the medium of *DEGS2* KO keratinocytes. A second possibility is that the PHS-containing CERs were produced *via de novo* synthesis with 2-hydroxy acyl-CoA as a precursor. In the first step of *de novo* sphingolipid synthesis, non-hydroxy acyl-CoA is condensed with serine to produce 3-ketodihydrosphingosine, followed by reduction to DHS. If 2-hydroxy acyl-CoA is used instead of non-hydroxy acyl-CoA in the first step, PHS is produced in the second reaction, and this is then converted to PHS-CER by CER synthase (34). However, this possibility is also unlikely, because PHS-CER levels were not increased by FA2H overproduction (Fig. 4*E*). A third possibility, which we consider the most likely, is the production of PHS-CERs by a hydroxylase other than DEGS2. DEGS1 shows no hydroxylation activity (Fig. 6*B*), despite its high sequence similarity to DEGS2. The second hydroxylase is therefore not DEGS1.

Besides DEGS2, there are other bifunctional enzymes exhibiting both desaturase and hydroxylase activities in plants and fungi (35, 36). A reaction model for these two activities of bifunctional enzymes has been proposed (36, 37). In this model, the reaction is initiated by an enzyme-bound iron-oxo species extracting a hydrogen atom from the substrate to form a carbon radical. When the hydrogen atom of the adjacent carbon is withdrawn from this radical intermediate, the desaturation reaction occurs. Alternatively, when this radical intermediate recombines with an oxygen atom bound to the active site, the hydroxylation reaction occurs. Although the factors that determine which reaction proceeds have not yet been elucidated, it is thought that the positioning of the substrate in the active site is involved. In this study, we found that the desaturase and hydroxylase activities of DEGS2 are higher toward DHS-CERs containing LCFAs and VLCFAs, respectively (Figs. 6 and 7). This difference in substrate specificity for FA chain length may be due to subtle changes in the positioning of the substrate in the active site based on its FA chain length.

PHS-CERs are abundant in epithelial tissues such as epidermis, stomach, small intestine, and kidney (Figs. 3 and 5). Since these epithelial cells are in contact with the outside, it is important that they have a strong barrier function. Studies measuring membrane permeability using model membranes composed of SPH-CER or PHS-CER have shown that membranes composed of PHS-CERs have lower membrane permeability and thus stronger barrier function than those composed of SPH-CERs (38). Since PHS-CERs have one more hydroxyl group than SPH-CERs, they can form more hydrogen bonds with neighboring lipids. The majority of the PHS-CERs in the epithelial tissues contained VLCFAs (Figs. 3 and 5). In addition, the anterior stomach and esophagus had more t20:0 PHS-CERs than other tissues (Fig. 7). These longer FA and/or LCB moieties of PHS-CERs may strengthen the hydrophobic interaction.

In the present study, we reveal that DEGS2 preferentially produces PHS-CERs containing VLCFAs. Furthermore, we suggest the existence of a pathway other than DEGS2 by which PHS-CERs are generated. In the future, it will be necessary to elucidate this unknown pathway and the importance of PHS-containing CERs in permeability barrier functioning in human epithelial tissues.

## Experimental procedures

### Mice

C57BL/6J mice were purchased from Sankyo Laboratory Services. *Degs2* KO mice were created *via* homologous recombination by introducing a neomycin resistance gene into exon 2 of *Degs2*. Mice were housed under a temperature of 23 ± 1 °C and humidity of 50 ± 5% with a 12-h light/dark cycle and free access to a standard chow diet (PicoLab Rodent Diet 20; LabDiet) and water, under specific pathogen-free conditions. The animal experiments were approved by the institutional animal care and use committee of Hokkaido University.

### Cells, growth conditions, and transfection

HAP1 cells (American Type Culture Collection) (39) and HEK 293T cells (RIKEN BioResource Research Center) were cultured in Iscove’s modified Dulbecco’s medium (Thermo Fisher Scientific) and Dulbecco’s modified Eagle’s medium (D6429; Merck), respectively, both containing 10% fetal bovine serum, 100 units/ml penicillin, and 100 μg/ml streptomycin (Merck) at 37 °C under 5% CO_2_. Transfections were performed using Lipofectamine Transfection Reagent with PLUS Reagent (Thermo Fisher Scientific), according to the manufacturer’s instructions.

Human immortalized keratinocytes (NHEK/SVTERT3-5), purchased from Evercyte, were cultured in CnT-Prime Epithelial Culture Medium (CELLnTEC, Bern) on dishes coated with 0.3% collagen (Nitta Gelatin) at 37 °C under 5% CO_2_. Transfections were performed using ViaFect Transfection Reagent (Promega), according to the manufacturer’s instructions. Keratinocyte differentiation *via* three-dimensional culture was performed as follows. Keratinocytes (5 × 10^5^ cells/ml) suspended in 400 μl differentiation medium (CnT-Prime Epithelial 3D Barrier Medium [CELLnTEC] containing 25 μg/ml ascorbic acid) were seeded on an insert lined with polyethylene terephthalate membrane (pore size 0.4 μm; catalog No. 353095; Corning), which had been placed onto a 24-well dish containing 500 μl of differentiation medium. After 24 h of incubation at 37 °C, the medium inside the insert was removed. Thereafter, cells were cultured for 14 days, changing only the medium outside the insert every 2 days.

### Permeability barrier assays

Toluidine blue staining, TEWL measurement, and hematoxylin/eosin staining were performed as previously described (16, 40). The lucifer yellow assay was performed as follows. The esophagus and anterior stomach of 6-month-old WT and *Degs2* KO mice were treated with 1 mM of lucifer yellow CH dilithium salt (Merck) in PBS (pH 7.4) for 3 h at 4 °C, washed three times with PBS, fixed with 3.7% of formaldehyde in 0.1 M sodium phosphate buffer (pH 7.4) for 24 h at 4 °C, equilibrated in 30% sucrose, and frozen in Tissue-Tek Optimal Cutting Temperature Compound (Sakura Finetek). Sagittal frozen sections (25 μm) were then prepared on a cryostat (CM3030 S, Leica Biosystems) and counterstained with 1 μg/ml of 4′,6-diamidino-2-phenylindole for nucleus staining. Fluorescence images were captured using a Leica DM5000B microscope equipped with a DFC295 digital color camera (Leica Microsystems).

### Plasmids

The human *DEGS1* and *DEGS2* genes were amplified from human kidney and lung cDNA (Takara Bio), respectively, *via* PCR using the following primers: *DEGS1*, 5′-GGATCCATGGGGAGCCGCGTCTCGCGGGAAG-3′ and 5′-TTACTCCAGCACCATCTCTCCTTTTTG-3′; and *DEGS2*, 5′-GGATCCATGGGCAACAGCGCGAGCCGCAGCG-3′ and 5′-TCACAGACCATCTTTTGCCAGCCTG-3′. The amplified genes were first cloned into the TA cloning vector pGEM-T Easy (Promega) and then transferred to pEFh-3× FLAG-1, which is a vector for expressing a protein with a 3× FLAG tag at the N-terminus (7), producing pEFh-3× FLAG-DEGS1 and pEFh-3× FLAG-DEGS2. The pCE-puro 3× FLAG-SPTLC1, pCE-puro 3× FLAG-SPTSSB, and pCE-puro HA-SPTLC3 plasmids have been described previously (15), as has the pCE-puro 3× FLAG-FA2H plasmid (41).

### Construction of KO cells

*DEGS1* KO HAP1 cells and *DEGS2* KO keratinocytes were generated using the CRISPR/Cas9 system as follows. We used the all-in-one CRISPR/Cas9 vector pYU417, which contains Cas9 D10A mutant nuclease (Cas9 nickase), the guide RNA cloning cassette, the enhanced green fluorescent protein gene, and the puromycin *N*-acetyltransferase gene (42). Two sequences of 20 bases adjacent to the protospacer-adjacent motif sequences in exon 2 of each gene were selected as guide RNAs. A pair of oligonucleotides corresponding to each guide RNA (*DEGS1*, 5′-CATTGCTTTGCAGTTGCCAAGTTTTTT-3′/5′-TTGGCAACTGCAAAGCAATGCGGTG-3′ and 5′-ATGTTTGCTAATCTTCCTATGTTTTTT-3′/5′-ATAGGAAGATTAGCAAACATCGGTG-3′; *DEGS2*, 5′-GCACCAGCACCGCCCACTTGGTTTT-3′/5′-CAAGTGGGGCGGTGCTGGTGCCGGTG-3′ and 5′-GCAGATGCTGGGCCTGCTGGGCGTTTTTT-3′/5′-GCCAGCAGGCCAGCATCTGCCGGTG-3′) were annealed and cloned into the pYU417 plasmid, generating pYU-DEGS1 and pYU-DEGS2 plasmids. After 24 h of transfection of HAP1 cells or human immortalized keratinocytes with the pYU-DEGS1 and pYU-DEGS2, respectively, puromycin (2.0 μg/ml final concentration) was added to the respective media (HAP1, Iscove’s modified Dulbecco’s medium; human immortalized keratinocytes, KGM-2 Keratinocyte Growth Medium [Lonza]) and cultured for another 24 h (HAP1) or 48 h (human immortalized keratinocytes). Human immortalized keratinocytes were cultured for an additional 2 days by replacing the medium with puromycin-free CnT-Prime Epithelial Culture Medium. Cells were then subcultured on new dishes at low cell densities to form colonies. After isolating the colonies, we prepared the genomic DNAs and analyzed them for the *DEGS1* or *DEGS2* gene sequence. We obtained a clone with a 33 bp deletion in exon 2 to use as *DEGS1* KO cells and two clones to use as *DEGS2* KO keratinocytes (KO 1, 49 bp deletion and 25 bp deletion; KO 2, 36 bp deletion and 2 bp deletion; all in exon 2). Two keratinocyte clones that did not have mutations were also obtained and used as controls (controls 1 and 2).

### *d*_7_-DHS labeling assay

*DEGS1* KO HAP1 cells cultured in six-well dishes were transfected with pEFh-3× FLAG-1, pEFh-3× FLAG-DEGS1, or pEFh-3× FLAG-DEGS2 plasmid. After 24 h transfection, cells were labeled with 2 μM *d*_7_-DHS (Avanti Polar Lipids) for 4 h. After the cells had been collected using a scraper, they were suspended in 150 μl of water, of which 50 μl was used for protein quantification and the remaining 100 μl was used for lipid extraction. Protein quantification was performed using the Pierce BCA Protein Assay Kit (Thermo Fisher Scientific) according to the manufacturer’s instructions. Lipid extraction was performed as follows. To the 100 μl cell suspension, we added 375 μl chloroform/methanol (1:2, v/v) and 2.5 pmol each of the internal standards (*N*-palmitoyl(*d*_9_)-D-*erythro*-SPH [*d*_9_-SPH-CER]; *N*-palmitoyl(*d*_9_)-D-*ribo*-PHS [*d*_9_-PHS-CER]; *N*-palmitoyl(*d*_9_)-D-*erythro*-DHS [*d*_9_-DHS-CER], LIPIDMAPS Mass Spec Internal Standard, Ceramide/Sphingoid Internal Standard Mix II [all from Avanti Polar Lipids]) and mixed vigorously. To hydrolyze the ester bonds in the glycerolipids, we incubated the samples with 12.5 μl of 3 M KOH at 37 °C for 1 h. After neutralization with 13 μl of 3 M formic acid, we added 125 μl chloroform and 125 μl water and mixed vigorously. The mixture was separated into two layers by centrifugation (20,400*g*, room temperature, 3 min), and the organic phase (lower layer) was collected and dried. The lipids thus prepared were suspended in 100 μl chloroform/methanol (1:2, v/v), and 5 μl was subjected to LC-MS/MS analysis as described below.

### Lipid analyses

Lipid extraction from differentiated keratinocytes in a three-dimensional culture was performed as follows. After the cells were washed with 500 μl of PBS, the insert membranes were cut out with a scalpel and transferred to test tubes. To these samples, we added 375 μl of chloroform/methanol (1:2, v/v) and *d*_9_-labeled CER/acyl-CER standards (*d*_9_-SPH-CER, 10 pmol; *d*_9_-DHS-CER, 5 pmol; *d*_9_-PHS-CER, 2.5 pmol; *N*-(26-oleoyloxy(*d*_9_) hexacosanoyl) D-*erythro*-SPH [*d*_9_-acyl-SPH-CER], 10 pmol [Avanti Polar Lipids]) and mixed vigorously. After sonication for 1 min, the samples were centrifuged (2400*g*, room temperature, 3 min). The pellet was subjected to protein quantification. The supernatant was mixed with 125 μl of chloroform and 125 μl of water sequentially. The samples were centrifuged (2400*g*, room temperature, 3 min), and the lower layer was collected and dried. The lipids thus prepared were suspended in 100 μl chloroform/methanol (1:2, v/v), and 5 μl was subjected to LC-MS/MS analysis as described subsequently. For SPH-CER measurement only, the samples were diluted 100-fold, and 5 μl was subjected to LC-MS/MS analysis.

Lipid extraction from mouse epidermis was conducted as follows. The skin was removed from postnatal day 0 mice, suspended in PBS, incubated at 55 °C for 5 min, and separated into dermis and epidermis. About 1.5 mg of epidermis was transferred to a tube containing zirconia beads, suspended in 450 μl chloroform/methanol (1:2, v/v), and crushed (4500 rpm, 4 °C, 1 min) using a Micro Smash MS-100 (Tommy Seiko). The solutions were collected, 450 μl of chloroform/methanol (1:2, v/v) was added to the remaining pellets, and the sample was crushed again. The solutions were collected and combined with the previously collected solutions, to which *d*_9_-labeled CER standards (*d*_9_-SPH-CER, 50 pmol; *d*_9_-DHS-CER, 100 pmol; and *d*_9_-PHS-CER, 12.5 pmol) were added. The solutions were divided into two samples, one of which was subjected to alkali treatment and the subsequent neutralization reaction as described previously (for SPH-CER, PHS-CER, and DHS-CER measurement), and the other was used without alkali treatment (for acyl-CER measurement). Chloroform and water were added to the samples for phase separation. After centrifugation, the organic phase was collected and dried. The lipids thus obtained were suspended in 100 μl of chloroform/methanol (1:2, v/v). Samples were diluted 660-fold (for SPH-CER measurement) or 33-fold (for the other CER classes measured) with chloroform, and 5 μl of each sample was subjected to LC-MS/MS analysis.

Lipid extraction from HEK 293T cells was performed as follows. Cells were collected using a scraper, then suspended in 150 μl of water, of which 50 μl was used for protein quantification and the remaining 100 μl was used for lipid extraction. To extract the lipids, we added 375 μl of chloroform/methanol (1:2, v/v) and 2.5 pmol each of the internal standards (*N*-(2′-(*R*)-hydroxypalmitoyl(*d*_9_)) D-*erythro*-SPH [2-OH *d*_9_-SPH-CER; Avanti Polar Lipids] and *d*_9_-PHS-CER) to 100 μl of cell suspension and mixed vigorously. The samples were then alkali-treated, neutralized, phase-separated, and dried as above. The lipids were suspended in 100 μl of chloroform/methanol (1:2, v/v), and 5 μl was subjected to LC-MS/MS analysis.

Lipid extraction from mouse tissues other than epidermis was performed as follows. Eleven tissues (brain, lung, heart, skeletal muscle, spleen, kidney, liver, small intestine, large intestine, testis, and stomach), six tissues (kidney, esophagus, anterior stomach, posterior stomach, small intestine, and large intestine), or two tissues (esophagus and anterior stomach) were collected from female C57BL/6J mice, respectively. Approximately 5 mg of tissues were transferred to tubes containing zirconia beads and mixed with 450 μl chloroform/methanol (1:2, v/v) and *d*_9_-labeled CER standards (*d*_9_-PHS-CER [5 pmol] for the 11 tissues; or *d*_9_-SPH-CER, *d*_9_-DHS-CER, and *d*_9_-PHS-CER [20 pmol each] for the six and two tissues). The tissues were crushed with a Micro Smash MS-100 and subjected to alkali treatment, neutralization, and phase separation as described above. The organic phase was collected and dried. The lipids were dissolved in 1.0 ml chloroform/methanol (1:2, v/v) and diluted to appropriate concentrations, and 5 μl was subjected to LC-MS/MS analysis.

### LC-MS/MS analyses

The LC-MS/MS analyses were performed using ultra-high-performance LC coupled with an electrospray ionization tandem triple quadrupole mass spectrometer (Xevo TQ-S; Waters) as described previously (43). A reversed-phase column ACQUITY UPLC CSH C18 column (particle size 1.7 μm, diameter 2.1 mm, length 100 mm; Waters) was used for liquid chromatography separation. Ionization was performed by electrospray ionization, and positive ions of CERs, acyl-CERs, SMs, and HexCERs were detected by MS/MS in multiple reaction monitoring (MRM) mode, where the *m*/*z* values of precursor and product ions specific to each ion were set for detection at Q1 and Q3. The MRM settings (*m*/*z* values for precursor and product ions and collision energy values) for unlabeled CERs and acyl-CERs were as described previously (15), whereas those for *d*_7_-labeled CERs, SMs, and HexCERs are shown in Tables S1–S3.

### Immunoblotting

Immunoblotting was performed as described previously (44). Anti-FLAG monoclonal antibody (M2; 1.85 μg/ml; Merck), anti-HA monoclonal antibody (3F10; 1000-fold dilution; Merck), and anti-GAPDH monoclonal antibody (5A12; 0.5 μg/ml; Fujifilm Wako Pure Chemical) were used as primary antibodies. Anti-mouse IgG HRP-conjugated F(ab′)_2_ fragment (1/7500 dilution; Cytiva) and anti-rat IgG HRP-conjugated F(ab′)_2_ fragment (1/1000 dilution; Cytiva) were used as the secondary antibodies. Labeling was detected using Western Lightning Plus-ECL (PerkinElmer Life Sciences).

### Imiquimod-induced psoriasis model

Cream containing 50 mg of imiquimod (Beselna cream; Mochida Pharmaceutical) and the control hydrophilic cream (Nikko cream; Nikko Pharmaceutical) were applied to both sides of the right and left ears, respectively, of 2- to 3-month-old mice every day for 4 days. On the fifth day, ear thickness was measured and total RNA was prepared from the ears. Total RNA preparation and quantitative real-time RT-PCR were performed as described previously (17). The primers used were as follows: *Il17a*, 5′-CCAGAAGGCCCTCAGACTACCTCAA-3′ and 5′-AGCATCTTCTCGACCCTGAAAGTGA-3′; *Il23a*, 5′-AATAATGTGCCCCGTATCCAGTGTG-3′ and 5′-GTCTCCCGGGGGTGATCCTCTGGCT-3′; *Gapdh*, 5′-GAACGGGAAGCTCACTGGCATGGGCC-3′ and 5′-TGTCATACCAGGAAATGAGCTTGAC-3′.

## Data availability

All data generated or analyzed during this study are contained within the article.

## Supporting information

This article contains supporting information.

## Conflict of interest

The authors declare no conflicts of interest in regard to this manuscript.
