# Supplementary material for: Bifunctional DEGS2 has higher hydroxylase activity toward substrates with very-long-chain fatty acids in the production of phytosphingosine ceramides
Source: J Biol Chem. 2023 Mar 11;299(4):104603. doi: 10.1016/j.jbc.2023.104603 (PMC10140171; doi:10.1016/j.jbc.2023.104603)
Supplement: Supporting Table S1 [file mmc1.docx]

**Table S1.** MRM settings for detection of *d*_7_-labeled CERs in LC-MS/MS analyses

| *d*_7_-labeled CER | Species | Precursor ions (Q1) | | Product ion (Q3) | Collision energy (eV) |
| --- | --- | --- | --- | --- | --- |
|  |  | [M–H_2_O + H]^+^ | [M + H]^+^ |  |  |
| SPH-CER | *d_7_*-d18:1/C16:0 | 527.6 |  | 271.3 | 20 |
| SPH-CER | *d_7_*-d18:1/C18:0 | 555.6 |  | 271.3 | 20 |
| SPH-CER | *d_7_*-d18:1/C20:0 | 583.6 |  | 271.3 | 20 |
| SPH-CER | *d_7_*-d18:1/C22:0 | 611.6 |  | 271.3 | 25 |
| SPH-CER | *d_7_*-d18:1/C24:1 | 637.7 |  | 271.3 | 30 |
| SPH-CER | *d_7_*-d18:1/C24:0 | 639.7 |  | 271.3 | 30 |
| SPH-CER | *d_7_*-d18:1/C26:0 | 667.7 |  | 271.3 | 30 |
| PHS-CER | *d_7_*-t18:0/C16:0 |  | 563.7 | 307.3 | 25 |
| PHS-CER | *d_7_*-t18:0/C18:0 |  | 591.7 | 307.3 | 25 |
| PHS-CER | *d_7_*-t18:0/C20:0 |  | 619.7 | 307.3 | 25 |
| PHS-CER | *d_7_*-t18:0/C22:0 |  | 647.8 | 307.3 | 30 |
| PHS-CER | *d_7_*-t18:0/C24:1 |  | 673.8 | 307.3 | 30 |
| PHS-CER | *d_7_*-t18:0/C24:0 |  | 675.8 | 307.3 | 30 |
| PHS-CER | *d_7_*-t18:0/C26:0 |  | 703.8 | 307.3 | 30 |
