# Supplementary material for: Bifunctional DEGS2 has higher hydroxylase activity toward substrates with very-long-chain fatty acids in the production of phytosphingosine ceramides
Source: J Biol Chem. 2023 Mar 11;299(4):104603. doi: 10.1016/j.jbc.2023.104603 (PMC10140171; doi:10.1016/j.jbc.2023.104603)
Supplement: Supporting Table S2 [file mmc2.docx]

**Table S2.** MRM settings for detection of *d*_7_-labeled HexCERs in LC-MS/MS analyses

| *d*_7_-labeled HexCER | Species | Precursor ions (Q1) | | Product ion (Q3) | Collision energy (eV) |
| --- | --- | --- | --- | --- | --- |
|  |  | [M–H_2_O + H]^+^ | [M + H]^+^ |  |  |
| SPH-HexCER | *d_7_*-d18:1/C16:0 | 689.6 |  | 271.3 | 40 |
| SPH-HexCER | *d_7_*-d18:1/C18:0 | 717.6 |  | 271.3 | 40 |
| SPH-HexCER | *d_7_*-d18:1/C20:0 | 745.6 |  | 271.3 | 40 |
| SPH-HexCER | *d_7_*-d18:1/C22:0 | 773.6 |  | 271.3 | 40 |
| SPH-HexCER | *d_7_*-d18:1/C24:1 | 799.7 |  | 271.3 | 40 |
| SPH-HexCER | *d_7_*-d18:1/C24:0 | 801.7 |  | 271.3 | 40 |
| SPH-HexCER | *d_7_*-d18:1/C26:0 | 829.7 |  | 271.3 | 40 |
| PHS-HexCER | *d_7_*-t18:0/C16:0 |  | 725.7 | 307.3 | 40 |
| PHS-HexCER | *d_7_*-t18:0/C18:0 |  | 753.7 | 307.3 | 40 |
| PHS-HexCER | *d_7_*-t18:0/C20:0 |  | 781.7 | 307.3 | 40 |
| PHS-HexCER | *d_7_*-t18:0/C22:0 |  | 809.7 | 307.3 | 40 |
| PHS-HexCER | *d_7_*-t18:0/C24:1 |  | 835.8 | 307.3 | 40 |
| PHS-HexCER | *d_7_*-t18:0/C24:0 |  | 837.8 | 307.3 | 40 |
| PHS-HexCER | *d_7_*-t18:0/C26:0 |  | 865.8 | 307.3 | 40 |
