# Supplementary material for: Bifunctional DEGS2 has higher hydroxylase activity toward substrates with very-long-chain fatty acids in the production of phytosphingosine ceramides
Source: J Biol Chem. 2023 Mar 11;299(4):104603. doi: 10.1016/j.jbc.2023.104603 (PMC10140171; doi:10.1016/j.jbc.2023.104603)
Supplement: Supporting Table S3 [file mmc3.docx]

**Table S3.** MRM settings for detection of *d*_7_-labeled SMs in LC-MS/MS analyses

| *d*_7_-labeled SM | Species | Precursor ion (Q1) | Product ion (Q3) | Collision energy (eV) |
| --- | --- | --- | --- | --- |
| SPH-SM | *d_7_*-d18:1/C16:0 | 710.6 | 184.1 | 60 |
| SPH-SM | *d_7_*-d18:1/C18:0 | 738.6 | 184.1 | 60 |
| SPH-SM | *d_7_*-d18:1/C20:0 | 766.6 | 184.1 | 60 |
| SPH-SM | *d_7_*-d18:1/C22:0 | 794.6 | 184.1 | 60 |
| SPH-SM | *d_7_*-d18:1/C24:1 | 820.7 | 184.1 | 60 |
| SPH-SM | *d_7_*-d18:1/C24:0 | 822.7 | 184.1 | 60 |
| SPH-SM | *d_7_*-d18:1/C26:0 | 850.7 | 184.1 | 60 |
| PHS-SM | *d_7_*-t18:0/C16:0 | 728.7 | 184.1 | 60 |
| PHS-SM | *d_7_*-t18:0/C18:0 | 756.7 | 184.1 | 60 |
| PHS-SM | *d_7_*-t18:0/C20:0 | 784.7 | 184.1 | 60 |
| PHS-SM | *d_7_*-t18:0/C22:0 | 812.7 | 184.1 | 60 |
| PHS-SM | *d_7_*-t18:0/C24:1 | 838.8 | 184.1 | 60 |
| PHS-SM | *d_7_*-t18:0/C24:0 | 840.8 | 184.1 | 60 |
| PHS-SM | *d_7_*-t18:0/C26:0 | 868.8 | 184.1 | 60 |
